# The influence of rutin and chlorogenic acid on oxidative stress and in vivo fertility: Evaluation of the quality and antioxidant status of post‐thaw semen from Azari water buffalo bulls

**DOI:** 10.1002/vms3.1548

**Published:** 2024-08-19

**Authors:** Tohid Mohammadi, Mohammadreza Hosseinchi Gharehaghaji

**Affiliations:** ^1^ Department of Basic Science, Faculty of Veterinary Medicine Urmia Branch, Islamic Azad University Urmia Iran

**Keywords:** chlorogenic acid, rutin, sperm cryo‐resistance, thawed buffalo sperm

## Abstract

**Background:**

The vulnerability of buffalo sperm to cryoinjury necessitates the improvement of sperm cryo‐resistance as a critical strategy for the widespread use of assisted reproductive technologies in buffalo.

**Objectives:**

The main aim of the present study was to evaluate the effects of different concentrations of rutin and chlorogenic acid (CGA) on buffalo semen quality, antioxidant activity and fertility during cryopreservation.

**Methods:**

The semen was collected and pooled from the 3 buffaloes using an artificial vagina (18 ejaculations). The pooled sperm were divided into nine different groups: control (Tris‐based extender); 0.4, 0.6, 0.8 and 1 mM rutin (rutin + Tris‐based extender); and 50, 100, 150 and 200 µM CGG (CGA + Tris‐based extender). Sperm kinematics, viability, hypo‐osmotic swelling test, mitochondrial activity, antioxidant activities and malondialdehyde (MDA) concentration of frozen and thawed buffalo sperm were evaluated. In addition, 48 buffalo were finally inseminated, and pregnancy was rectally determined 1 month after insemination.

**Results:**

Compared to the control group, adding R‐0.4, R‐0.6, CGA‐100 and CGA‐150 can improve total and progressive motility, motility characteristics, viability, PMF and DNA damage in buffalo sperm. In addition, the results showed that R‐0.4, R‐0.6, CGA‐50, CGA‐100 and CGA‐150 increased total antioxidant capacity, catalase, glutathione peroxidase and glutathione activities and decreased MDA levels compared to the control group. Furthermore, it has been shown that adding 150 µM CGA and 0.6 mM rutin to an extender can increase in vivo fertility compared to the control group.

**Conclusions:**

In conclusion, adding rutin and CGA to the extender improves membrane stability and in vivo fertility of buffalo sperm by reducing oxidative stress.

## Graphical abstract

Adding rutin and chlorogenic acid to semen extender enhances sperm motility, improves PMF, reduces sperm viability damage and boosts sperm antioxidant capacity. Thus, their inclusion can enhance the quality of frozen and thawed buffalo semen. This research also indicates that rutin and chlorogenic acid may be beneficial in enhancing in vivo fertility.

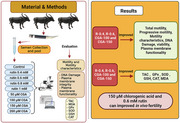

## 1 INTRODUCTION

The Azari ecotype is characterized by its tiny size, black skin and notable attributes like a high‐quality milk production rate, elevated fat content and extended milking time each year (pour Azary et al., 2004; Borghese & Mazzi, 2005). The widespread use of artificial insemination (AI) technology has significantly increased the consumption rate of buffalo semen. AI and cryopreservation of sperm are crucial in conserving buffalo genetic resources within this particular livestock species (Ramazani et al., 2023a). Additionally, the cryopreservation of gametes in the banking system provides a reliable and effective means of protecting biodiversity and endangered species. Cryopreservation, which involves storing sperm in liquid nitrogen at an extremely low temperature of −196°C, can preserve sperm. According to Li et al. (2023), this phenomenon extends the lifespan of the cells. AI may be conducted with prolonged or frozen‐thawed semen. Cryogenic damage can result from various factors, including osmotic stress, cold shock, intracellular ice crystal formation, excessive reactive oxygen species (ROS) production and alterations in antioxidant defence mechanisms. As part of regular physiological processes, ROS and antioxidant enzymes are produced harmoniously. However, excessive ROS formation coupled with a decrease in the activity of antioxidant defence mechanisms leads to reduced sperm motility and viability, DNA damage and protein denaturation (Archana et al., 2023).

Buffalo sperm are prone to membrane damage, which may be due to the increased content of long‐chain polyunsaturated fatty acids (PUFAs) in the sperm membrane. This high concentration makes the membrane more sensitive to lipid peroxidation (LPO) when ROS are present (Yuan et al., 2023). Buffalo semen is known to have several antioxidant systems that can lower ROS levels and mitigate internal cellular damage (Gao et al., 2017; Luo et al., 2023; Turaja et al., 2019). However, the cryopreservation process poses a challenge as semen has low levels of these antioxidants, leaving the endogenous defence system unable to combat this stress (Ansari et al., 2012). So, it is essential to have an outside, robust antioxidant system to stop or lessen the effects of LPO and boost sperm function, which keeps fertility at its best (Izanloo et al., 2021, 2022; Ramazani et al., 2023b; Sheikholeslami et al., 2020; Soleimanzadeh et al., 2020; Soleimanzadeh & Saberivand, 2013; Soleimanzadeh et al., 2014). Numerous studies have investigated the influence of different extenders on the cryopreservation of buffalo semen to improve the quality of buffalo semen during storage. However, it has been found that results vary depending on the specific type and concentration of antioxidants used (Dorostkar et al., 2012; Iqbal et al., 2016; Kumaresan et al., 2006; Luo et al., 2023; Mostafa et al., 2019; Swami et al., 2017).

Rutin, a flavonoid glycoside often referred to as vitamin P or purple quercetin, is derived from several natural sources, including *Ruta graveolens*, tobacco, jujube, apricot, orange, tomato and buckwheat. Several scientific studies have shown evidence of the antioxidant activity and anti‐inflammatory effects of rutin (Cândido et al., 2018; Liu et al., 2018). The effectiveness of rutin in removing ROS directly by donating electrons to free radicals has been shown in other studies (Ghiasi et al., 2012). According to recent studies by Jamalan et al. (2016) and Mehfooz et al. (2018), there is evidence that rutin may have a potentially protective effect on male reproductive function both in vivo and in vitro; according to the findings of Xu et al. (2020), the addition of rutin to the cryopreservation extender enhances the antioxidant defence mechanism and protects boar sperm from ROS attack. In another study by Najafi et al. (2023), rutin could improve epididymal sperm quality in sheep after the cryopreservation procedure.

Chlorogenic acid (CGA) is a phenolic acid compound abundant in many food sources, such as coffee and tea (Meng et al., 2013; Venditti et al., 2015). CGA has been shown to have remarkable biological properties, such as its ability to act as an antioxidant (Mussatto et al., 2011), exert anti‐inflammatory effects (Guo et al., 2015) and exhibit anti‐tumour effects (Granado‐Serrano et al., 2007). Castro et al. (2018) discovered that CGA could scavenge free radicals and inhibit the progression of oxidative reactions in vitro. Previous research has shown that incorporating 4.5 mg/mL CGA into boar semen can improve the quality of insemination doses, particularly for storage periods exceeding 24 hr (Pereira et al., 2014). In a study by Namula et al. (2018), it was shown that adding 100 µM CGA during the process of freezing boar semen resulted in a significant improvement in various sperm properties, including motility, viability and plasma membrane functionality (PMF).

However, a thorough review of the existing literature has shown that the influence of rutin and CGA on buffalo sperm has not yet been investigated in vivo or in vitro. Therefore, the main aim of this research was to examine the influence of rutin and CGA on maintaining the quality of buffalo sperm after cryopreservation when used as supplements in the Tris‐based extender.

## 2 MATERIALS AND METHODS

### 2.1 Collection and processing of the semen

All animals used in this study were healthy and housed under identical conditions. The artificial vagina technique was used to collect sperm twice a week. Three Iranian water buffalo bulls (*Bubalus bubalis*) with 7 weeks of proven fertility produced 18 ejaculations. Only semen samples subjected to a quality assessment that satisfied specific requirements were used for the research. Specimens with a concentration of more than 500 × 10^6^ cells/mL, a volume between 2 and 6 mL, total motility of more than 65% and abnormal morphology of less than 20% per ejaculation were considered normal and included in the study. The animals involved in the study were well cared for, and they received the same treatment. The regulations of the Animal Ethics Committee carried out the study Islamic Azad University, Iran.

Merck and Sigma supplied all necessary chemicals. The present study used a typical Tris‐based extender as the base extender for semen dilution (Ramazani et al., 2023a). Semen samples (*n* = 3) from each replication were pooled, divided into nine parts and diluted to a final concentration of 15 × 10^6^ spermatozoa/mL (Soleimanzadeh, Talavi et al., 2020) with one of the following extenders. The extender was used as a control group (C). In addition to the control group, eight additional groups were formed by incorporating different concentrations of antioxidants into the extender. Rutin (Xu et al., 2020) and CGA (Namula et al., 2018) were added to the Tris‐Base extender at concentrations of 0.4, 0.6, 0.8 and 1 mM (R‐0.4, R‐0.6, R‐0.8 and R‐1) and 50, 100, 150 and 200 µM (CGA‐50, CGA‐100, CGA‐150 and CGA‐200), respectively. The frozen samples were defrosted after 1 week and then prepared for analysis by thawing at 38°C in a water bath (Soleimanzadeh, Talavi et al., 2020).

### 2.2 Semen analysis

#### 2.2.1 Motility and motion parameters

Researchers can gain important information about the quality of the semen sample and its potential impact on male fertility by using a computer‐assisted semen analysis (CASA) system to assess semen motility characteristics. A CASA (Test Sperm 3.2; video test) system was used to determine sperm motility parameters. Some motility parameters were measured using the system. These were total motility, progressive motility, curvilinear velocity (VCL), straight‐line velocity (VSL), average path velocity (VAP), straightness, linearity (LIN), amplitude of lateral head displacement and beat‐cross frequency (BCF). Each analysis required 10 µL of thawed sperm, and a minimum of 500 sperm were examined in 5 microscopic areas (Table 1).

**TABLE 1:** Parameter settings for the computer‐assisted semen analysis (CASA).

Parameter	Setting
**Frame rate**	60 Hz
**Duration of capture**	1 s
**Stage temperature rate**	37°C
**Minimum cell size**	5 pixels
**Cell size**	5 pixels
**Minimum contrast**	80
**Cell intensity**	70 pixels
**Chamber type**	Slide‐coverslip (22 × 22 mm^2^)
**Volume per slide**	7 µL
**Chamber depth**	≈20 µm
**Minimum number of field analysis**	500 cells
**sample dilution**	20 × 10^6^
**Image type**	Phased contrast

#### 2.2.2 DNA damage evaluation

A low pH technique called acridine orange (AO) staining was used to check for DNA damage and find broken double‐stranded DNA segments in sperm chromatin (Narayana et al., 2002). The semen sample was formed into a thick swab, which was then fixed in Carnoy's fixative (a 1:3 mixture of methanol and acetic acid) for 2 h. The smear was then extracted and air‐dried for 5 min at room temperature. The smear was then incubated for 5 min at 4°C in the dark with a stock solution of 1 mg AO and 1000 mL of distilled water (Soleimanzadeh, Kian et al., 2020). Sperm were examined with a fluorescence microscope (Model GS7, Nikon Co.). Yellow or red fluorescent sperm were considered damaged and aberrant, suggesting possible DNA fragmentation.

#### 2.2.3 Sperm plasma membrane functionality

The hypo‐osmotic swelling test is a helpful tool for assessing the function of the sperm membrane. For this test, a small amount of semen is diluted in a hypo‐osmotic solution containing fructose and sodium citrate and then incubated at 37°C. The plasma membrane of the sperm is evaluated after incubation with a contrast phase microscope (Olympus, BX41) at 400× magnification. The percentage of sperm with intact membranes is often calculated by counting 200 straight or curled sperm (Khan & Ijaz, 2007; Ramazani et al., 2023a).

#### 2.2.4 Viability

Eosin–nigrosine staining was used to assess viability, according to a World Health Organization (WHO) (1999). The pigments eosin and nigrosine were formed in distilled water. One volume of semen was combined with two volumes of 1 % eosin, and the combination was then examined at 400× magnification with a light microscope (Olympus, BX41). Viable sperm remain colourless, but non‐viable sperm turn red due to eosin staining (Ramazani et al., 2023a).

### 2.3 Assessment of enzymatic antioxidant activity

Six straws were used for the biochemical examination of semen, one for each extender. Thawed sperm of 120 µL was taken and centrifuged at 1600 × *g* and 25°C for 5 min. To extract the sperm enzymes, the supernatant was removed and then treated with 360 µL of 1% Triton X‐100. The mixture was subjected to precipitation for 20 min before being centrifuged at 4000 × *g* for 30 min at 25°C (Ramazani et al., 2023b).

The levels of total antioxidant capacity (TAC), glutathione peroxidase (GPx), superoxide dismutase (SOD), catalase (CAT), glutathione (GSH) and malondialdehyde (MDA) in semen samples were analysed using kits from Navand Salamat Company. The TAC levels were reported in mmol/L, whereas the GPx levels were in mU/mL. On the other hand, the SOD, CAT and GSH activities in the seminal plasma were expressed in U/mL, the GPx activity was given in mU/mL and the MDA values were shown in nmol/mL (Ramazani et al., 2023b). For 2,2‐diphenyl‐1‐picrylhydrazyl (DPPH) radical scavenging evaluation, in a cuvette that held 970 mL of mixed methanol, 5 mL (10 mM) of DPPH radicals were added. The absorbance at 517 nm (A517) was measured after incubating this mixture at 20°C for 3 min. After adding and mixing 25 mL of each sample and 25 mL of a 9.50 M acetonitrile solution as a negative control, the mixture was incubated at 20°C for 3 min. It was then determined how much the A517 value had fallen as a result of the breakdown of the DPPH radicals (Chrzczanowicz et al., 2008).

### 2.4 Fertility rate following artificial insemination

We conducted a fertility study in vivo on a selected group of 48 adult wheeled buffalo aged 5–8 years with no known reproductive issues, similar nutrition (Table 2) and body condition scores of 2–3 (Escalante et al., 2013). To compare fertility based on post‐thaw results, we divided the buffalo into three groups: the control group, the CGA‐150 (150 µM CGA) group and the R‐0.6 (0.6 mM rutin) group (best groups). After an experienced inseminator performed inseminations, each buffalo underwent inseminations by an inseminator, and a rectal pregnancy diagnosis was made at least 60 days after insemination (Ramazani et al., 2023a).

**TABLE 2:** Composition of concentrate mixtures fed to adult wheeled buffalo.

Ingredients	Lactating cows (kg)
Corn	470.00
Soybean meal	150.00
Full‐fat soy flour	80.00
Fish meal	50.00
Bran	120.00
Calcium carbonate	12.00
Dicalcium phosphate	4.00
Molasses	40.00
Salt	8.00
Saponification of fat powder	50.00
Sodium bicarbonate	13.00
Vitamins^a^	1.00
Minerals^b^	2.00
Total	1000.00

^a^ Vitamin premix formula: vitamin A, 16,000,000 IU; vitamin D3, 6,000,000 IU; and vitamin E, 100,000 mg.

^b^ Mineral premix formula (per kg): ferrous sulphate, 60.00 g; manganese sulphate, 50.00 g; copper sulphate, 12.50 g; sodium selenite, 0.40 g; zinc sulphate, 50.00 g; and cobalt carbonate, 0.15 g.

### 2.5 Statistical analysis

Study data were analysed using SPSS software (version 26.0, IBM). A one‐way analysis of variance was performed to determine if there were significant differences between the groups studied. Tukey's post hoc test was then used to determine which groups were significantly different from each other. A *p*‐value of ≤0.05 was considered statistically significant.

## 3 RESULTS

### 3.1 Motility of the sperm and movement features

Based on the results presented in Table 3, it appears that groups R‐0.4, R‐0.6, R‐1, CGA‐50, CGA‐100 and CGA‐150 in terms of sperm total and progressive motility provide better outcomes compared to the control group (*p* ≤ 0.05; Table 3). CASA evaluated these factors after the freezing process. Furthermore, there were no significant differences in the R‐0.8 and CGA‐200 groups compared to the control group (*p* > 0.05; Table 3).

**TABLE 3:** Effects of different concentrations of rutin (R) and chlorogenic acid (CGA) on buffalo sperm total and progressive at the post‐thaw stage of cryopreservation.

Analysis	Control	R‐0.4	R‐0.6	R‐0.8	R‐1	CGA‐50	CGA‐100	CGA‐150	CGA‐200
Total motility (%)	54.03 ± 1.54^fg^	61.05 ± 1.20^cd^	65.18 ± 1.36^ab^	57.32 ± 1.42^ef^	59.03 ± 1.19^de^	61.49 ± 1.73^cd^	64.59 ± 1.51^bc^	68.24 ± 1.14^a^	53.72 ± 1.30^g^
Progressive motility (%)	22.19 ± 1.40^f^	30.82 ± 1.89^cd^	34.03 ± 1.65^ab^	25.92 ± 1.15^ef^	27.45 ± 1.37^de^	30.35 ± 1.63^cd^	33.54 ± 1.90^bc^	37.89 ± 1.78^a^	23.45 ± 1.20^f^

*Note*: Values are expressed as mean ± SEM. R‐0.4: Tris‐based extender + rutin (0.4 mM); R‐0.6: Tris‐based extender + rutin (0.6 mM); R‐0.8: Tris‐based extender + rutin (0.8 mM); R‐1: Tris‐based extender + rutin (1 mM); CGA‐50: Tris‐based extender + chlorogenic acid (50 µM); CGA‐100: Tris‐based extender + chlorogenic acid (100 µM); CGA‐150: Tris‐based extender + chlorogenic acid (150 µM); CGA‐200: Tris‐based extender + chlorogenic acid (200 µM). Different superscripts within the same row demonstrate significant differences (*p *≤ 0.05).

Table 4 shows the characteristics of buffalo sperm motility rates. According to Table 4, the groups receiving R‐0.4, R‐0.6, R‐1, CGA‐50, CGA‐100 and CGA‐150 showed increased VAP, LIN and BCF compared to the control group (*p* ≤ 0.05; Table 4). However, adding R‐0.8 and CGA‐200 to the extender in VAP and LIN and CGA‐200 in BCF had no significant effects compared to the control group (*p* ≤ 0.05, Table 4). VCL analysis showed that groups R‐0.4, R‐0.6, R‐0.8, R‐1, CGA‐50, CGA‐100 and CGA‐150 had higher values than the control group (*p* ≤ 0.05, Table 4). In addition, the R‐0.4, R‐0.6, CGA‐50, CGA‐100 and CGA‐150 groups performed better than the control group on VSL analysis (*p* ≤ 0.05, Table 4).

**TABLE 4:** Mean (±SEM) sperm motility characteristics of frozen–thawed buffalo semen after the addition of different concentrations of rutin (R) and chlorogenic acid (CGA) in the semen extender.

Analysis	Control	R‐0.4	R‐0.6	R‐0.8	R‐1	CGA‐50	CGA‐100	CGA‐150	CGA‐200
VAP (µm/s)	21.30 ± 1.32^e^	27.74 ± 1.97^bcd^	29.45 ± 1.87^bc^	23.92 ± 1.81^e^	24.01 ± 1.69^de^	26.80 ± 1.73^cd^	30.15 ± 1.60^ab^	33.20 ± 1.36^a^	21.40 ± 1.49^e^
VCL (µm/s)	31.54 ± 1.57^e^	39.10 ± 0.48^bc^	42.59 ± 1.57^b^	34.40 ± 1.65^d^	36.47 ± 1.07^cde^	40.24 ± 1.20^b^	42.27 ± 1.03^b^	46.77 ± 1.69^a^	32.85 ± 1.70^e^
VSL (µm/s)	16.08 ± 1.61^c^	21.41 ± 1.76^b^	25.45 ± 1.80^a^	17.09 ± 1.17^c^	17.51 ± 1.40^c^	20.37 ± 1.48^b^	25.13 ± 1.95^a^	27.05 ± 1.56^a^	15.91 ± 1.27^c^
LIN (%)	42.19 ± 1.15^f^	51.73 ± 1.36^c^	54.38 ± 1.39^b^	44.72 ± 1.76^ef^	47.53 ± 1.28^de^	49.78 ± 1.67^cd^	56.94 ± 1.12^ab^	58.27 ± 1.71^a^	42.10 ± 1.33^f^
ALH (µm/s)	2.47 ± 0.10^a^	2.45 ± 0.12^a^	2.46 ± 0.11^a^	2.45 ± 0.13^a^	2.47 ± 0.10^a^	2.48 ± 0.14^a^	2.48 ± 0.13^a^	2.50 ± 0.16^a^	2.47 ± 0.14^a^
STR (%)	72.05 ± 1.32^a^	72.39 ± 1.47^a^	72.85 ± 1.25^a^	73.05 ± 2.30^a^	72.40 ± 1.19^a^	72.66 ± 1.51^a^	72.26 ± 1.50^a^	73.19 ± 1.83^a^	72.19 ± 1.59^a^
BCF (Hz)	4.61 ± 0.19^e^	5.30 ± 0.16^cd^	6.32 ± 0.34^b^	4.83 ± 0.15^d^	5.07 ± 0.23^d^	5.39 ± 0.29^cd^	5.82 ± 0.26^c^	7.05 ± 0.30^a^	4.60 ± 0.24^e^

*Note*: R‐0.4: Tris‐based extender + rutin (0.4 mM); R‐0.6: Tris‐based extender + rutin (0.6 mM); R‐0.8: Tris‐based extender + rutin (0.8 mM); R‐1: Tris‐based extender + rutin (1 mM); CGA‐50: Tris‐based extender + chlorogenic acid (50 µM); CGA‐100: Tris‐based extender + chlorogenic acid (100 µM); CGA‐150: Tris‐based extender + chlorogenic acid (150 µM); CGA‐200: Tris‐based extender + chlorogenic acid (200 µM); VAP: average path velocity; VCL: curvilinear velocity; VSL: straight‐line velocity; LIN: linearity; ALH: amplitude of lateral head displacement; BCF: beat‐cross frequency; STR: straightness. Different superscripts within the same row demonstrate significant differences (*p *≤ 0.05).

### 3.2 Plasma membrane functionality, DNA damage and sperm viability

**FIGURE 1:**
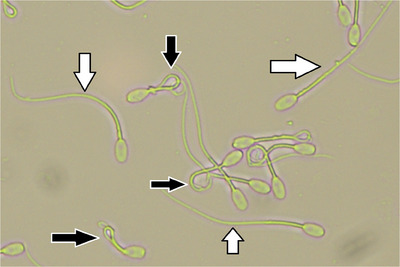
Sperm plasma membrane (PM) functionality. White arrows – buffalo spermatozoa with straight tails (nonfunctional PM); black arrows – buffalo spermatozoa with coiled tails (functional PM) (400×).

**FIGURE 2:**
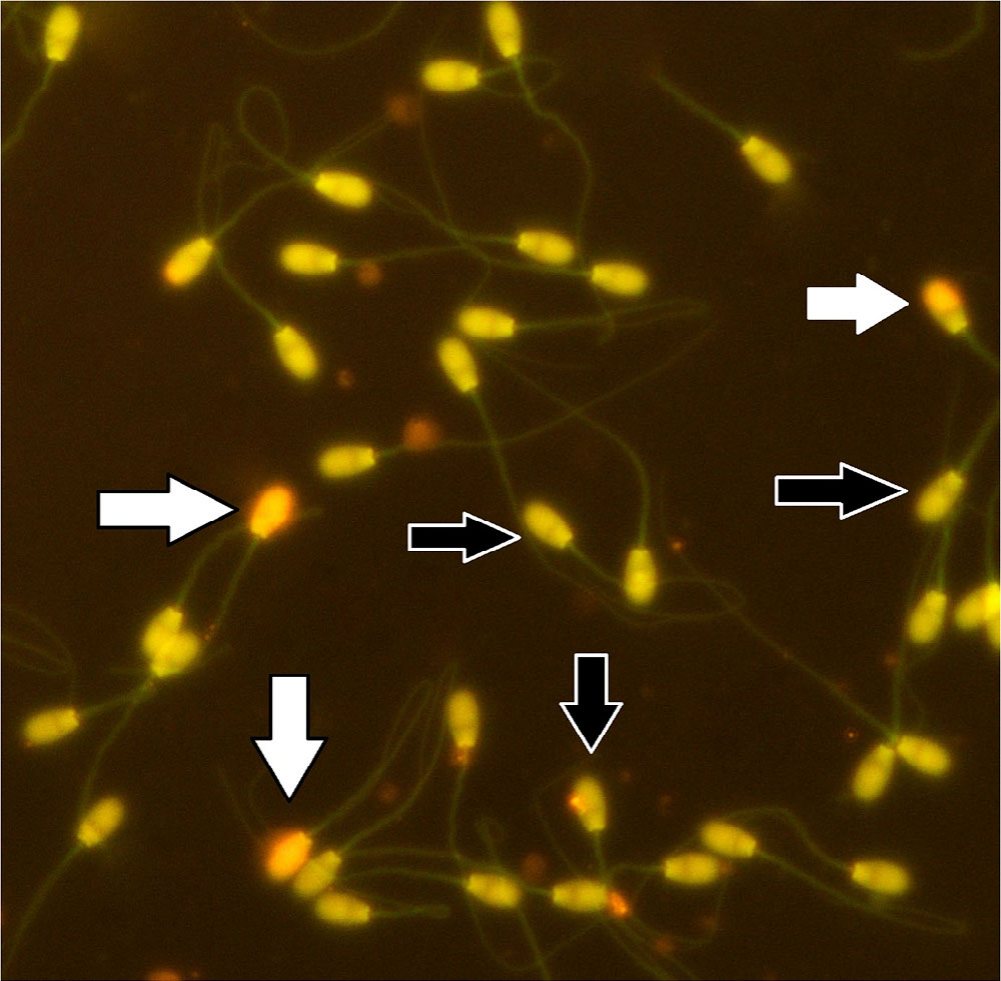
Sperm DNA damage. Yellow arrows – normal spermatozoa (green); white arrows – DNA damaged spermatozoa (yellow‐red) (acridine orange, 400×).

**FIGURE 3:**
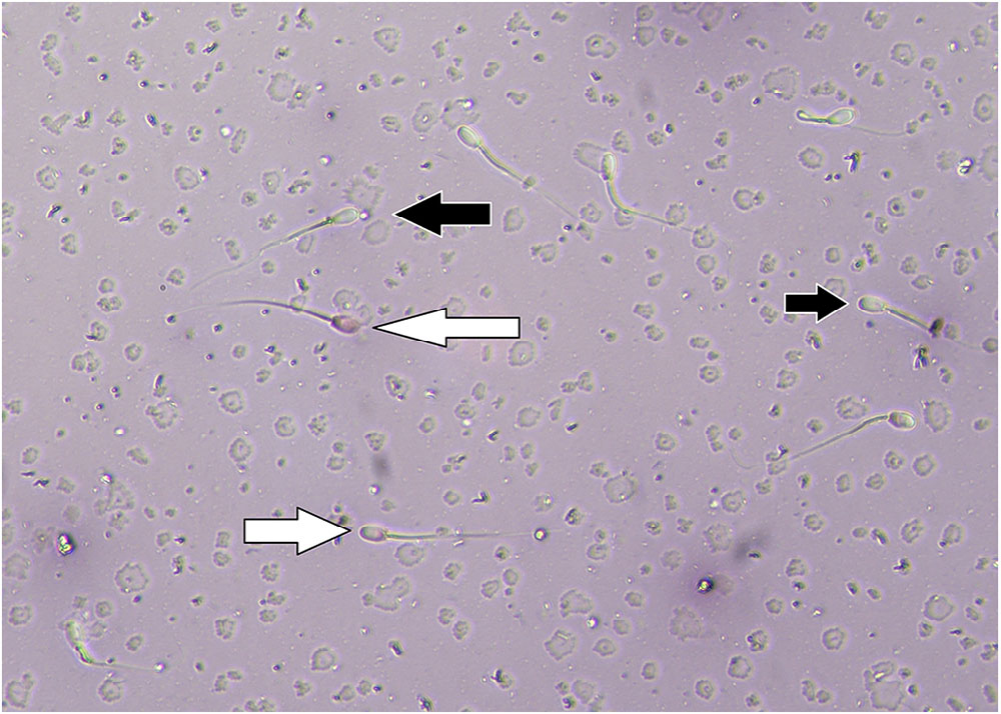
Sperm plasma membrane (PM) integrity. Black arrows – viable spermatozoa (colourless); white arrow – dead spermatozoa (red) (eosin/nigrosine, 200×).

Table 5 presents the PMF results of cryopreserved buffalo sperm. Compared to the control group, more sperm in the R‐0.4, R‐0.6, CGA‐50, CGA‐100 and CGA‐150 treatment groups had intact plasma membranes (*p* ≤ 0.05, Figure 1; Table 5). Moreover, Table 5 shows that the DNA integrity of the buffalo sperm was affected by different R and CGA values. Compared to the control group, adding R‐0.4, R‐0.6, R‐1, CGA‐50, CGA‐100 and CGA‐150 improved sperm DNA integrity (*p* ≤ 0.05, Figure 2; Table 5). In addition, the study found that sperm viability values were highest in the R‐0.4, R‐0.6, CGA‐100 and CGA‐150 groups compared to the control group (*p* ≤ 0.05, Table 5). However, there were no significant differences in the viability percentage between the R‐0.8, R‐1, CGA‐50 and CGA‐200 groups and the control group (*p *> 0.05; Figure 3; Table 5).

**TABLE 5:** Effect of different concentrations of rutin (R) and chlorogenic acid (CGA) supplementation on DNA damage, viability and plasma membrane functionality (PMF) (mean ± SEM) in frozen–thawed buffalo sperm.

Analysis	Control	R‐0.4	R‐0.6	R‐0.8	R‐1	CGA‐50	CGA‐100	CGA‐150	CGA‐200
Viability (%)	63.30 ± 1.71^d^	68.94 ± 1.55^bc^	71.05 ± 1.47^ab^	64.46 ± 2.53^d^	64.05 ± 1.81^d^	66.78 ± 1.37^cd^	70.93 ± 1.43^ab^	73.42 ± 2.10^a^	63.91 ± 2.36^d^
DNA damage (%)	10.84 ± 0.26^a^	7.82 ± 0.32^d^	6.47 ± 0.29^e^	10.41 ± 0.40^a^	9.64 ± 0.35^b^	8.05 ± 0.31^c^	6.79 ± 0.28^e^	5.46 ± 0.34^f^	10.76 ± 0.41^a^
Sperm plasma membrane functionality (%)	58.19 ± 1.65^c^	64.15 ± 1.08^b^	68.51 ± 1.73^a^	59.82 ± 1.32^c^	62.30 ± 1.83^bc^	64.20 ± 1.72^b^	67.92 ± 1.57^ab^	70.85 ± 1.22^a^	59.22 ± 1.74^c^

*Note*: R‐0.4: Tris‐based extender + rutin (0.4 mM); R‐0.6: Tris‐based extender + rutin (0.6 mM); R‐0.8: Tris‐based extender + rutin (0.8 mM); R‐1: Tris‐based extender + rutin (1 mM); CGA‐50: Tris‐based extender + chlorogenic acid (50 µM); CGA‐100: Tris‐based extender + chlorogenic acid (100 µM); CGA‐150: Tris‐based extender + chlorogenic acid (150 µM); CGA‐200: Tris‐based extender + chlorogenic acid (200 µM). Different superscripts within the same row demonstrate significant differences (*p *≤ 0.05).

### 3.3 Analysis of antioxidant activities

**FIGURE 4:**
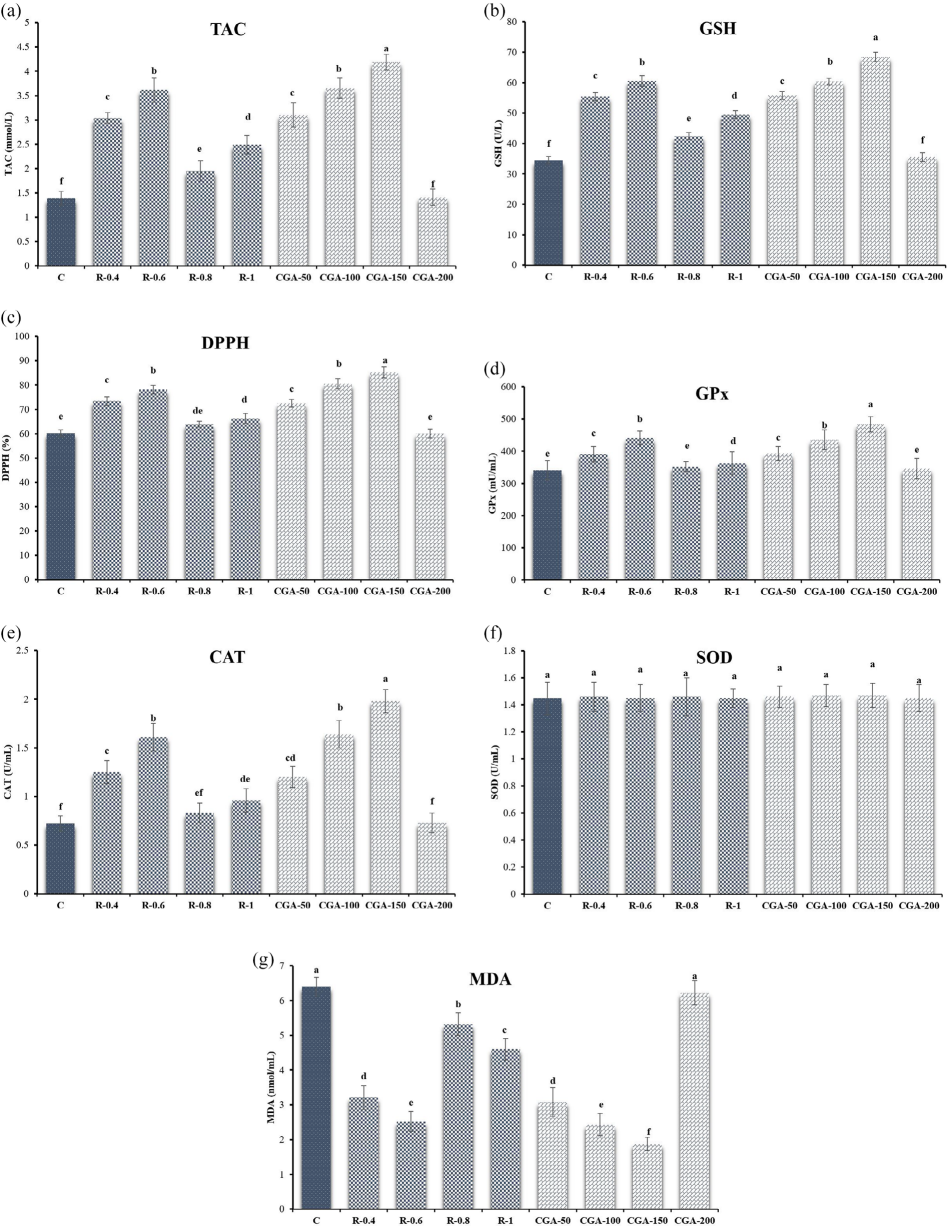
(a) Total antioxidant capacity (TAC); (b) glutathione (GSH) activities; (c) 2,2‐diphenyl‐1‐picrylhydrazyl (DPPH); (d) glutathione peroxidase (GPx); (e) catalase (CAT); (f) superoxide dismutase (SOD); (g) lipid peroxidation (MDA) of frozen–thawed buffalo semen after supplementation of different concentrations of rutin (R) and chlorogenic acid (CGA) to the semen extender. Control (c): Tris‐based extender without antioxidant; R‐0.4: Tris‐based extender + rutin (0.4 mM); R‐0.6: Tris‐based extender + rutin (0.6 mM); R‐0.8: Tris‐based extender + rutin (0.8 mM); R‐1: Tris‐based extender + rutin (1 mM); CGA‐50: Tris‐based extender + chlorogenic acid (50 µM); CGA‐100: Tris‐based extender + chlorogenic acid (100 µM); CGA‐150: Tris‐based extender + chlorogenic acid (150 µM); CGA‐200: Tris‐based extender + chlorogenic acid (200 µM). Different superscripts within the same row demonstrate significant differences (*p* ≤ 0.05; mean ± SEM).

Figure 4 shows the results for antioxidant activities (TAC, GPx, CAT, GSH, DPPH and MDA) in buffalo spermatozoa treated with different concentrations of R and CGA. The groups supplemented with R‐0.4, R‐0.6, R‐0.8, R‐1, CGA‐50, CGA‐100 and CGA‐150 showed significantly higher levels of TAC, GSH and DPPH scavenger compared to the control group (*p* ≤ 0.05, Figure 4A–C). At the same time, there was no significant difference between treated with CGA‐200 in TAC and GSH levels and treated with R‐0.8 and CGA‐200 in DPPH scavenger with the control group (*p *> 0.05; Figure 4A–C). The GPx and CAT activity analyses showed a significant increase in the R‐0.4, R‐0.6, R‐1, CGA‐50, CGA‐100 and CGA‐150 groups compared to the control group (*p* ≤ 0.05, Figure 4D,E). Notably, the addition of R‐0.8 and CGA‐200 did not affect the GPx and CAT levels compared to the control group (*p* > 0.05; Figure 4D,E). The superoxide dismutase scores showed no significant differences between the groups (*p* > 0.05; Figure 4F). MDA analysis revealed that adding R‐0.4, R‐0.6, R‐0.8, R‐1, CGA‐50, CGA‐100 and CGA‐150 decreased MDA levels compared to the control group (*p* ≤ 0.05, Figure 4G).

### 3.4 Analysis of fertility rate

Table 6 provides data on in vivo fertility test results. Regarding fertility, there was no significant difference between the R and CGA groups. However, the 150 µM CGA and 0.6 mM rutin groups had higher conception rates than the control group (*p* ≤ 0.05; Table 6).

**TABLE 6:** comparison of fertility rate of buffalo semen cryopreserved.

Extender	Inseminations	Pregnancy rate (%)
Control	16	68.75 (11/16)^b^
CGA‐150	16	87.50 (14/16)^a^
R‐0.6	16	81.25 (13/16)^a^

*Note*: CGA‐150: Tris‐based extender + chlorogenic acid (150 µM); R‐0.6: Tris‐based extender + rutin (0.6 mM); Different superscripts within the same column demonstrate significant differences (*p *≤ 0.05).

## 4 DISCUSSION

Antioxidants are frequently employed to enhance the quality of thawed sperm, as oxidative stress can degrade the quality of frozen sperm. Our research indicates that the use of R and CGA may significantly enhance frozen buffalo semen. Other research on buffalo semen has demonstrated that the addition of various antioxidants to frozen–thawed semen can improve semen quality parameters (Ashabi et al., 2023; Farjami et al., 2023; Jahangiri Asl et al., 2021; Salehi et al., 2023; Soleimanzadeh et al., 2018, 2017; Soleimanzadeh et al., 2020).

Sperm motility is a crucial predictor of fertility, although it is not the sole determinant. Other factors, such as sperm viability and the integrity of mitochondria and cell membranes, also play significant roles (Ahmed et al., 2016). Reduced sperm motility during chilled storage can be attributed to both mitochondrial membrane failure and plasma membrane disruption (Pagl et al., 2006). Numerous studies have demonstrated that adding antioxidants to semen extenders can yield several benefits. These include improved sperm morphology, increased motility, protection of the plasma membrane and a reduction in ROS synthesis in frozen–thawed sperm (Shahzad et al., 2016; Tariq et al., 2015). For instance, caffeic acid, an antioxidant, not only protects against oxidative damage and cold shock but can also enhance sperm motility after thawing (Hu et al., 2010). Our study further supports these findings. Extenders treated with rutin and CGA significantly enhanced post‐thaw sperm kinematic properties, including progressive and total motility. This aligns with a previous study by Aksu et al. (2017), which reported improved rat sperm motility and reduced abnormalities with rutin therapy. Additionally, it has been shown that rutin supplementation positively impacted parameters such as VSL, VCL and VAP in post‐thawed sperm (Xu et al., 2020). Najafi et al. (2023) conducted a study demonstrating that supplementing ram sperm with rutin boosted mitochondrial activity and inhibited apoptosis. Their findings suggest that rutin enhances sperm motility and viability by improving mitochondrial function. Rutin possesses antioxidant properties, which protect cells from oxidative damage caused by ROS. By reducing oxidative stress and supporting mitochondrial health, rutin enhances energy production within sperm cells, ultimately boosting sperm motility. Additionally, a study found that CGA can increase the total motility of boar sperm stored for 72 h (Rabelo et al., 2020). Furthermore, dietary supplements containing arachidic acid or butylated hydroxytoluene have also been shown to increase motility in frozen and thawed buffalo sperm (Ejaz et al., 2014; Ijaz et al., 2009).

Cryopreservation and thawing procedures induce LPO of PUFAs in sperm cell membranes, resulting in ultrastructural changes (Partyka et al., 2012). Excessive production of ROS during freezing and thawing disrupts sperm and seminal plasma antioxidant defences, impacting semen quality and fertilization ability (Peña et al., 2009). The sperm plasma membrane experiences heightened LPO, leading to membrane deterioration and increased permeability. This progressive permeability contributes to the depletion of intracellular antioxidant enzymes, ultimately impairing sperm function (Alvarez & Storey, 1995). Rutin treatment has been shown to reduce ROS formation, enhance viability and improve membrane integrity in sperm (Mata‐Campuzano et al., 2012; Najafi et al., 2023). Similarly, adding CGA to semen extenders improves sperm viability, motility and PMF after thawing (Namula et al., 2018). Our study supplemented the freezing extender with rutin and CGA, resulting in significantly increased sperm viability and PMF. These observations are consistent with a previous survey highlighting rutin's antioxidant activity and positive impact on mitochondrial function (Dudylina et al., 2019). Furthermore, antioxidant supplementation has enhanced post‐thaw viability in buffalo sperm (El‐Sheshtawy et al., 2008). Other studies suggest that royal jelly or green tea extract may also improve sperm viability in buffalo after preservation (Ahmed et al., 2020; Shahzad et al., 2016).

Research has demonstrated that the integrity of sperm chromatin may be compromised due to cryopreservation and thawing procedures (Anzar et al., 2002). Furthermore, the generation of ROS during cryopreservation leads to increased DNA damage (Kadirvel et al., 2009). Our investigation reveals that incorporating rutin and CGA into the semen extender can effectively reduce sperm DNA damage. Similarly, Dorostkar et al. (2012) found that adding sodium selenite to the extender mitigates DNA damage in buffalo sperm. Additionally, Topraggaleh et al. (2014) reported that including cysteine in the extender decreases DNA damage in buffalo sperm. Furthermore, Ejaz et al. (2014) demonstrated that adding arachidic acid to the extender improves the integrity of sperm chromatin in buffalo semen.

Cryopreservation and thawing procedures expose buffalo semen to cold shock and atmospheric oxygen, increasing the risk of LPO. This process damages the structural integrity of sperm membranes due to the heightened production of ROS (Ramazani et al., 2023a). Consequently, antioxidant enzyme activity decreases, and a negative correlation exists between sperm MDA levels, viability, PMF and overall motility. The plasma membrane experiences increased LPO during freezing and thawing, leading to membrane leakage, loss of integrity and depletion of intracellular antioxidant enzymes (Oldenhof et al., 2013). LPO occurs when partially reduced oxygen molecules oxidize membrane lipids (Ramazani et al., 2023b). Studies indicate that rutin enhances antioxidant capacity by activating CAT, GPx and superoxide dismutase in rat brain cells (Annapurna et al., 2013). Similarly, rutin improves antioxidant defences in cryopreserved boar semen, protecting against ROS attack (Xu et al., 2020). Khan et al. (2017) explored rutin's effects on sperm damage induced by ROS or LPO in rats. Our research supports the antioxidant benefits of R and CGA, as evidenced by increased TAC and reduced MDA levels. R and CGA enhance TAC, GPx, reduced GPx and CAT levels in buffalo semen after cryopreservation. Notably, R and CGA inhibit protein dephosphorylation during cryopreservation (Fu et al., 2018). Although antioxidants safeguard sperm during freezing, excessive doses may reduce effectiveness due to hypertonic extenders (Bucak et al., 2007). Additionally, taurine supplementation significantly enhances antioxidant levels in post‐preserved buffalo sperm (Reddy et al., 2010).

Research has revealed that alterations and damage can impact sperm fertility without necessarily affecting sperm motility. This suggests that sperm fertility may decline before other factors, such as motility, viability and sperm membrane functionality, exhibit noticeable changes (Chatterjee et al., 2001; Reddy et al., 2010). In a study by Xu et al. (2020), the addition of rutin to the boar semen extender resulted in improved cleavage and blastocyst rates after semen cryopreservation. Similarly, another study demonstrated that supplementing boar semen with CGA enhances in vitro fertilization outcomes (Namula et al., 2018). Our findings indicate that using 150 µM CGA and 0.6 mM rutin improves fertility in vivo compared to the control group. Additionally, other studies suggest that adding antioxidants to buffalo sperm extenders enhances fertility (Longobardi et al., 2017; Mohammadi et al., 2024; Ramazani et al., 2023a). Quercetin has also been found to enhance sperm fertility in vivo. Furthermore, adding cysteine during cryopreservation of buffalo sperm activates the antioxidant system, improves sperm motility and increases in vivo fertility (Iqbal et al., 2016).

## 5 CONCLUSION

In conclusion, the supplementation of rutin and CGA to the seminal fluid extender results in enhanced sperm motility, improved PMF, decreased impairment of sperm viability and heightened sperm antioxidant capability. Hence, incorporating rutin and CGA holds promise for ameliorating the quality of cryopreserved buffalo semen. This investigation further posits that rutin and CGA may be beneficial for enhancing in vivo fertility.

## AUTHOR CONTRIBUTIONS

Tohid Mohammadi was involved in the idea, design, data collecting, statistical analysis and paper preparation. Tohid Mohammadi and Mohammadreza Hosseinchi Gharehaghaji all contributed to the study's supervision as well as the manuscript's drafting. The final version was accepted for submission by all writers.

## CONFLICT OF INTEREST STATEMENT

None of the authors have any conflicts of interest to declare.

## FUNDING INFORMATION

This research has not been financially supported.

## ETHICS STATEMENT

The authors confirm the ethical policies of the journal, as noted on the journal's author guidelines page. The study was carried out in accordance with the regulations of the Animal Ethics Committee of Islamic Azad University, Iran (IR‐IAU‐2/37/29).

## Data Availability

The data that support the findings of this study are available on request from the corresponding author. The data are not publicly available due to privacy or ethical restrictions.
